# Alien leaf beetles (Coleoptera, Chrysomelidae) of European Russia and some general tendencies of leaf beetle invasions

**DOI:** 10.1371/journal.pone.0203561

**Published:** 2018-09-07

**Authors:** Andrzej O. Bieńkowski, Marina J. Orlova-Bienkowskaja

**Affiliations:** A.N. Severtsov Institute of Ecology and Evolution, Russian Academy of Sciences, Moscow, Russia; Chinese Academy of Agricultural Sciences Institute of Plant Protection, CHINA

## Abstract

Invasions of leaf beetles can cause tremendous economic consequences because some of these insects become major pests in invaded territories. We present the first inventory of alien Chrysomelidae of European Russia that appeared in the region in the 20th and 21st centuries (9 species) with analysis of the history of their invasions and detailed maps of distribution. This case study revealed some general tendencies of invasions of leaf beetles: (1) Recently, a dramatic increase in the rate of Chrysomelidae invasions is observed, which reflects the increase in international trade of living plants; (2) Alien leaf beetles can spread quickly, occupying almost all of Europe within several decades; (3) When the range of some leaf beetle species is quickly expanding, or when the species has been recorded established somewhere outside the native range, this species should be regarded as a potential invader worldwide. and (4) Alien leaf beetles usually occur on alien or cultivated plants, but some become naturalized in native communities. The specific information was the following. Two species native to the Mediterranean region, *Chrysolina americana* (feeds on *Rosmarinus* and *Lavandula*) and *Leptomona erythrocephala* (feeds on *Lotus corniculatus*) were recorded in European Russia for the first time. A polyphagous pest of floriculture *Luperomorpha xanthodera* native to China and Korea and a pest of soybeans *Medythia nigrobilineata* native to east Asia have been in the region since 2016. A pest of tobacco *Epitrix hirtipennis* native to North America has occurred since 2011. A pest of corn *Diabrotica virgifera* was intercepted at the border of Russia in 2011 but has not established. Three alien species have been in the region since the 20th century: *Zygogramma suturalis* introduced from North America for control of *Ambrosia*, *Phyllotreta reitteri* native to Afghanistan and Tajikistan and feeding on *Lepidium latifolium*, and the Colorado potato beetle *Leptinotarsa decemlineata*.

## Introduction

Major gaps in knowledge occur in regional alien floras and faunas, and data availability varies among regions [1]. In particular, alien insects of European Russia are poorly studied. Most of the information is published in local sources and remains unknown to a wider audience because the information is not included in international databases. We present the first inventory of alien leaf beetles of this territory.

Leaf beetles (Chrysomelidae (except Bruchinae), Megalopididae and Orsodacnidae) are a large group of phytophagous beetles (more than 36 000 species worldwide, with approximately 600 in European Russia) [2, 3]. Negative economic impact of invasions of these insects can be significant, because some of them, e.g., *Leptinotarsa decemlineata*, *Diabrotica virgifera* and *Epitrix papa*, are major pests of various crops [4]. Despite the impact, invasions of leaf beetles are poorly studied [5]. Before 2010, only 11 species alien to Europe had been recorded [6], and the information about invasions of leaf beetles to European Russia was restricted to *Leptinotarsa decemlineata* and *Zygogramma suturalis* [7–9].

Four alien species were first recorded in European Russia in 2011–2016: *Diabrotica virgifera*, *Epitrix hirtipennis*, *Luperomorpha xanthodera* and *Medythia nigrobilineata* [10–13]. Analysis of the ecology and dynamics of ranges of *Chrysolina eurina* (Frivaldszky, 1883) (Chrysomelinae) and *Lilioceris lilii* (Scopoli, 1763) (Criocerinae) reveals that they could represent archeoinvaders, i.e., alien species established in European Russia before the 20th century [14, 15]. Here, we present the first records of two alien species new to European Russia, *Leptomona erythrocephala* and *Chrysolina americana*, and a review of all other leaf beetles alien to this region. For each species, a database of records and a map of geographical distribution were compiled, and the following information was provided: data on host plants and other features of biology, description of native range, invasion history, possible vectors of dispersal and economic impact of establishment. This article is a part of the project "Alien beetles of European Russia."

## Materials and methods

Distribution of alien species was studied based on collecting by the authors in European Russia in 1988–2018 and examination of specimens from collections of ZIN (Zoological Institute of Russian Academy of Sciences, St. Petersburg, Russia), ZMMU (Zoological Museum of Moscow State University, Moscow, Russia), FNMS (Senckenberg Naturmuseum, Frankfurt am Main, Germany), HNHM (Hungarian Natural History Museum, Budapest, Hungary), MTD (Museum für Tierkunde, Dresden, Germany), NHMW (Naturhistorisches Museum, Wien, Austria), NMP (National Museum in Prague, Prague, Czech Republic), VNIIKR (Russian Plant Quarantine Center, Bykovo, Russia), BSU (Belgorod State University, Belgorod, Russia), OSU (Orel State University, Orel, Russia), BNR (Belogorie Nature Reserve, Borisovka, Russia), and 17 private collections in Russia: AB (A.O. Bieńkowski, Zelenograd), AK (A.G. Koval, St. Petersburg), AP (A.I. Prikhodko, Zelenograd), AR (A.B. Ruchin, Saransk), AU (A.S. Ukrainsky, Moscow), EI (E.V. Iljina, Makhachkala), GK (G.A. Korostov, Elista), MD (M.M. Dolgin, Syktyvkar), NN (N.E. Nikolaeva, Tver), NO (N.V. Okhrimenko, Krasnodar), PP (P.N. Petrov, Moscow), PR (P.V. Romantsov, St. Petersburg), RI (R.N. Ishin, Tambov), SM (S.A. Mosyakin, Simferopol), TM (T.A. Mogilevich, Zelenograd), VF (V.I. Filippov, Sochi), and YK (Y.N. Kovalenko, Moscow). Additionally, data from the Global Biodiversity Information Facility and 82 literature sources were used. We used the criteria of alien status and terminology used in invasion biology defined by Richardson et al. [16] and Blackburn et al. [17]. The maps of distribution of species were prepared using the program DIVA-GIS (http://diva-gis.org/download) [18]. These maps were based on GADM maps under a CC BY license, with permission from Robert Hijmans, owner of GADM (original copyright 2001).

In this study, we adhered to the system adopted in the Catalogue of Palaearctic Coleoptera [19–21]. We studied invasions of leaf beetles only in the narrow sense of the word, i.e., all subfamilies of Chrysomelidae, except seed beetles (Bruchinae), because seed beetles are very different from other Chrysomelidae in biology and ecology and have different vectors and trends of invasions [6]. Megalopididae and Orsodacnidae are also leaf beetles, but we did not consider these families because no alien species of these groups occur in European Russia. The species below are presented in reverse chronological order of their first records in European Russia.

### 2017 –*Leptomona erythrocephala* (Olivier, 1790) (Galerucinae)

#### Native range

**Fig 1:**
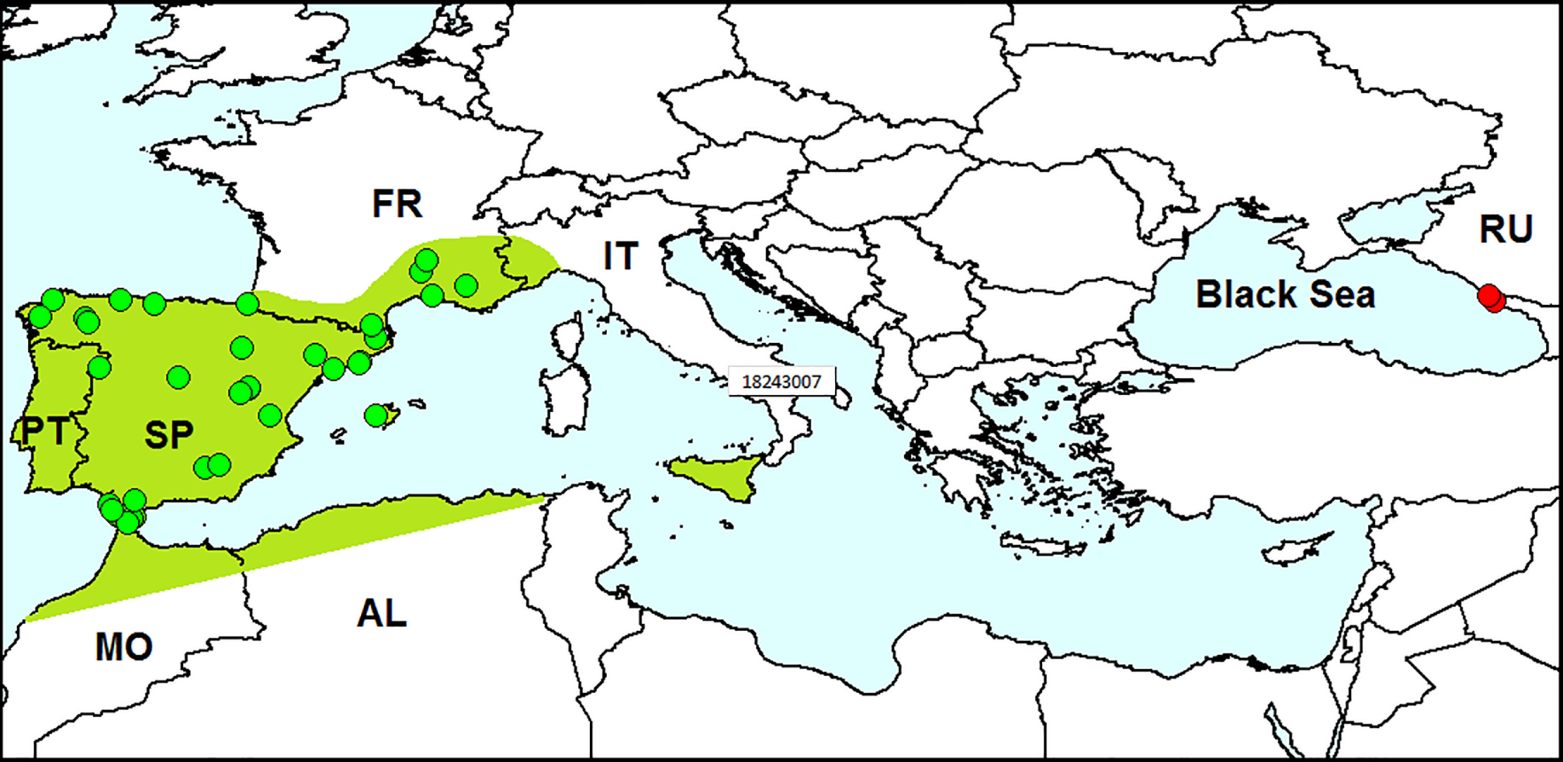
Distribution of *Leptomona erythrocephala*. *Red dots*–localities of specimens outside the species native range (original data); *green dots* on the *green background*–localities of specimens in the native species range. AL–Algeria, FR–France, IT–Italy, MO–Morocco, PT–Portugal, RU–Russia, SP–Spain. Database on localities of *L*. *erythrocephala* is provided in S1 Appendix.

*Leptomona erythrocephala* is native to mainland Spain, Mallorca, Portugal, south France, Sicily, Algeria and Morocco (Fig 1) [20, 22–24]. Junior synonym *Monolepta verticalis* Reitter, 1886 was described from Portugal [22]. *Leptomona erythrocephala* was also recorded from northern Italy, but this record is supposedly questionable [23].

#### Invasion history

This species was not previously recorded outside its native range.

#### Records in European Russia

In June 2017 and May 2018, we found *L*. *erythrocephala* in the northwest Caucasus, on the Black Sea coast. Ten specimens were sweep-netted in ruderal plants at the Imereti Resort, Adler District, Sochi (43°24′ N, 39°58′ E), and approximately 50 specimens were found on the ruderal plant *Lotus corniculatus* in Razdolnoe Vill., on the floodplain of the Bzugu River, Khosta District, Sochi (43°36′ N, 39°46′ E).

#### Taxonomy and identification

The genus *Leptomona* Bechyné, 1958 was distinguished by J. Bechyné from the large genus *Monolepta* Chevrolat, 1836 and includes four species: *L*. *erythrocephala* distributed in the Mediterranean region, *L*. *russica* (Gmelin, 1790) distributed on the steppes of eastern Europe and western Asia, *L*. *fulvicollis* (Jacoby, 1885) distributed in Japan and *L*. *subseriata* (Weise, 1887) distributed in east Siberia and the Far East. Only one species, *L*. *russica* has been recorded in European Russia [25].

**Fig 2:**
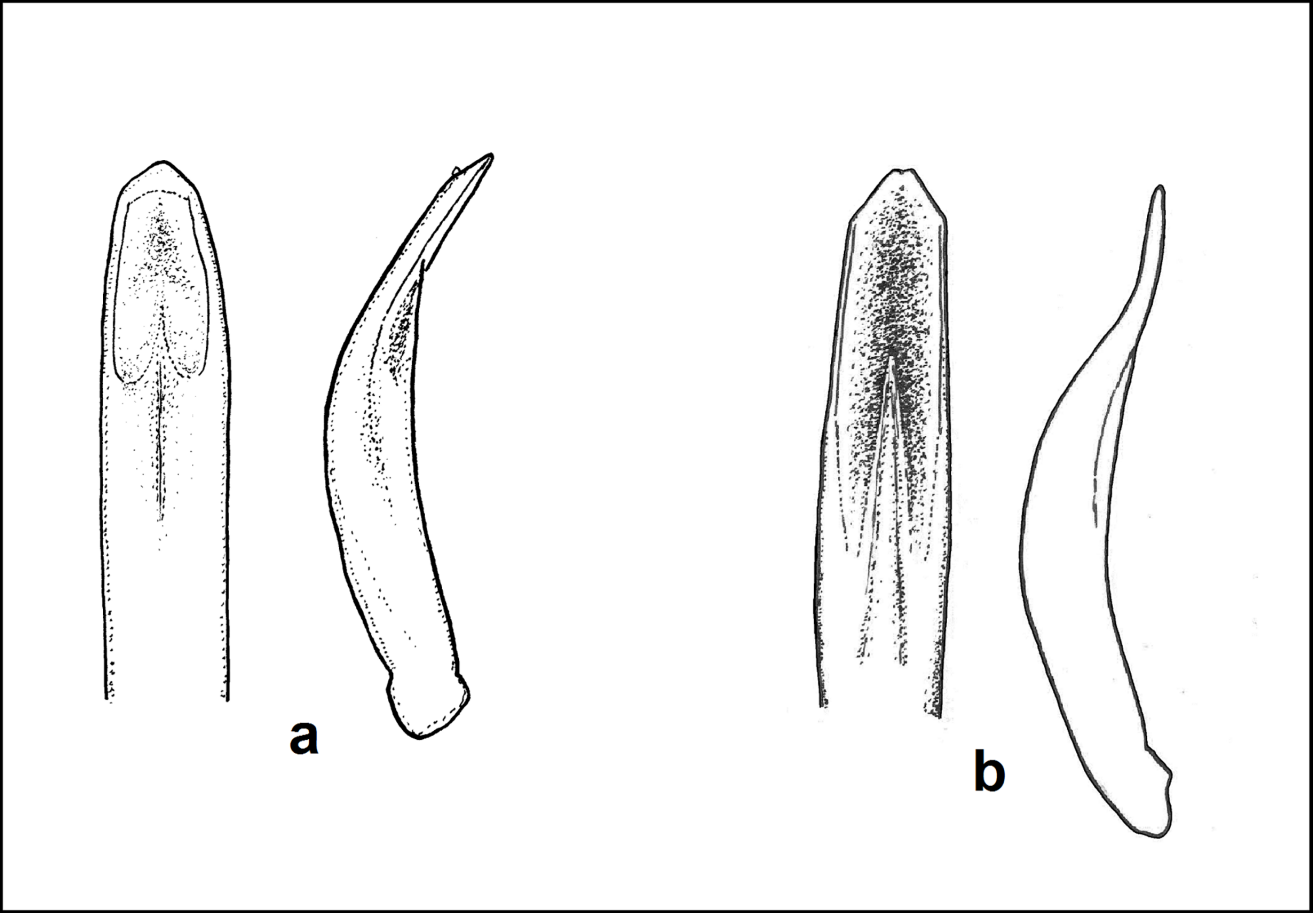
Aedeagus of *Leptomona erythrocephala* from Sochi (**a**) and *L*. *russica* (**b**).

*Leptomona erythrocephala* differs from other species of the genus by the shape of the aedeagus (Fig 2) and by the following characters: punctation of elytra entirely confused; pronotum covered with punctures that are slightly smaller than those on elytra; head, pronotum, prosternum, coxae, femora, tibiae and antennomeres 1–3 reddish, mesosternum brown, elytra blue, labrum, tarsi, metasternum, abdominal tergites and sternites black; hind wings reduced. Identification of specimens collected in Sochi (see S2 Appendix) was confirmed by their comparison with specimens of *L*. *erythrocephala* from ZIN collected in Spain.

#### Biology

This species in its native range feeds on *Polygonum* and is also recorded on *Fragaria* and *Potentilla reptans* [26, 27]. However, *L*. *russica* and representatives of the closely related genus *Monolepta* feed primarily on Fabaceae [28]. Based on our observations in nature, *L*. *erythrocephala* feeds on *Lotus corniculatus* (Fabaceae), which is a common ruderal plant in the city. Based on observations in a cage, we confirmed feeding on this plant. Ten specimens were placed in cage with the following Fabaceae plants collected in the same biotope: *Trifolium repens*, *Trifolium aureum*, *Gleditsia triacanthos* and *Lotus corniculatus*. The beetles fed only on *L*. *corniculatus*, gnawing on margins of leaves and petals.

#### Invasion status

Obviously, *Leptomona erythrocephala* is established in the region. First, many specimens were collected in the wild in two localities in 2017 and 2018. Second, the beetle feeds on a native plant.

#### Vector of dispersal

Sochi is more than 2000 km distant from the native range of the species; thus, natural spread was impossible. Unintentional introduction with planting material or soil was the most likely dispersal vector. The distance between the two localities in which *L*. *erythrocephala* was found in the Caucasus is 28 km. Because *L*. *erythrocephala* is a flightless beetle and its natural dispersal ability is restricted, the dispersal of *L*. *erythrocephala* in the region was likely connected with an unintentional introduction by man.

#### Economic impact

*Leptomona erythrocephala* is not regarded as a pest in its native range. However, special attention should be paid to trophic specialization of this species in the Caucasus, particularly if it becomes abundant. Note that *L*. *erythrocephala* is recorded to feed on *Fragaria* leaves [26], the representatives of the genera *Leptomona* and *Monolepta* feed on Fabaceae, and one of them, *Monolepta quadriguttata* (Motschulsky, 1860), is a serious pest of soybean [28, 29].

### 2016 –*Luperomorpha xanthodera* (Fairmaire, 1888) (Alticinae)

#### Native range

Rose flea beetle is native to China and the Korean Peninsula [21].

#### Invasion history

**Fig 3:**
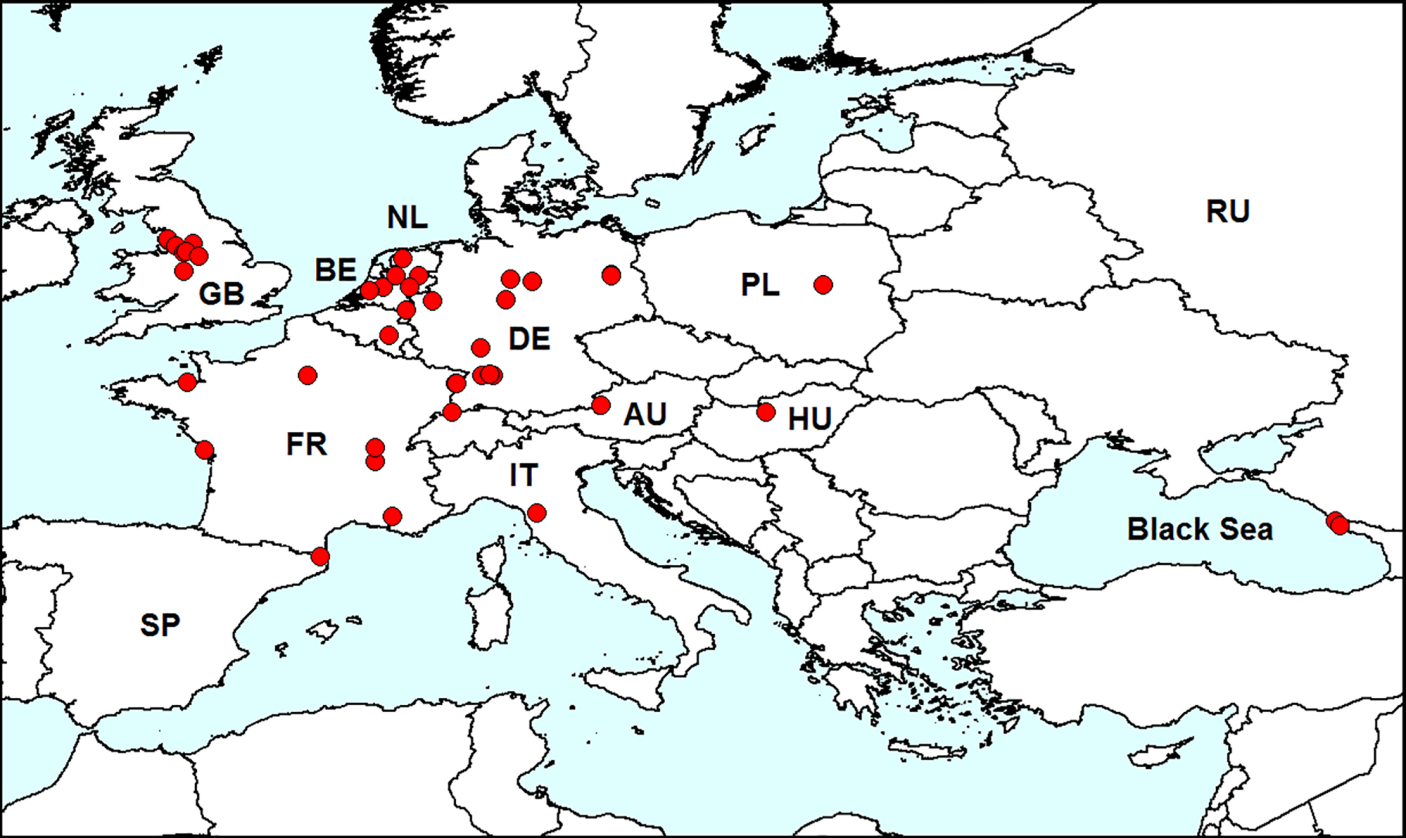
Distribution of *Luperomorpha xanthodera* in Europe. *Red dots*–localities of *L*. *xanthodera* in Europe. AU–Austria, BE–Belgium, DE–Germany, FR–France, GB–Great Britain, HU–Hungary, IT–Italy, NL–Netherlands, PL–Poland, RU–Russia, SP–Spain. Database on localities of *L*. *erythrocephala* is provided in S1 Appendix.

This pest was first found in Europe in 2003 on the British Islands (Fig 3) [30]. Then, *L*. *xanthodera* quickly spread to Italy [31], France [32], Germany, Switzerland [33], The Netherlands [34], Hungary [35], Austria [36], Poland [37], Belgium [38], Spain [39] and European Russia [12].

#### Records in European Russia

In 2016–2018, *L*. *xanthodera* was first recorded in European Russia in Sochi (Central District and Adler District) [12]. The beetles are usually on rose flowers and occur on ruderal vegetation in May–June.

#### Biology

*Luperomorpha xanthodera* is a polyphagous species. Adults feed on flowers of many plants (23 genera from 19 families), and larvae develop on roots of these plants [40]. In Sochi, adults of *L*. *xanthodera* feed on flowers of roses and citrus plants [12].

#### Vector of dispersal

*Luperomorpha xanthodera* was most likely unintentionally introduced as larvae on roots of imported seedlings or as adults transported as cargo stowaways in airplanes. Both vectors are possible in the region of Sochi. First, this city is close to the international airport. Second, mass planting with imported planting material was conducted in Sochi during the landscaping of the city in preparation for the Olympic games in 2014. Many other invasive pests were introduced and established in the city in this period [12].

#### Invasion status

*Luperomorpha xanthodera* is established in the region. Finds of numerous specimens in three subsequent years (2016–2018) in different localities indicated that a self-sustaining population exists in the wild and that the species is dispersing in the region.

#### Economic impact

Establishment of *L*. *xanthodera* in the south of European Russia could cause negative economic consequences, because the beetle is a pest of ornamental flowers.

### 2016 –*Medythia nigrobilineata* (Motschulsky, 1860) (Galerucinae)

#### Native range

**Fig 4:**
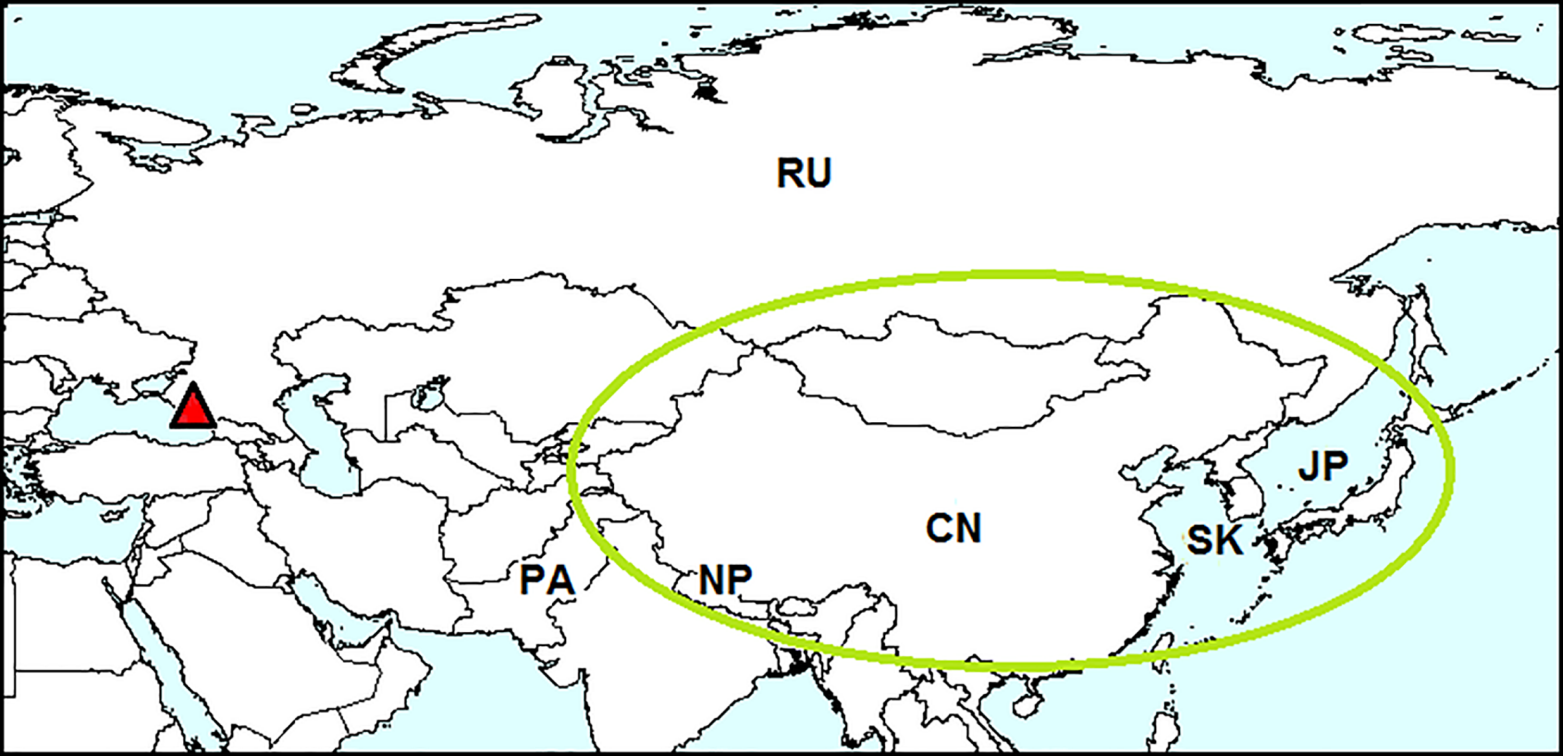
Distribution of *Medythia nigrobilineata*. *Red triangle*–locality of the first record outside the native range: Sochi. Native range is *circled with green*. CN–China, JP–Japan, NP–Nepal, PA–Pakistan, RU–Russia, SK–South Korea. Sources of information: [13, 20, 41–43].

Two-striped leaf beetle *Medythia nigrobilineata* (= *Paraluperodes suturalis nigrobilineatus*) is native to north China, Japan, Nepal, Pakistan, South Korea, east Siberia and the Russian Far East (Fig 4) [20, 41–43].

#### Invasion history

In 2016, this species was first found outside its native range, in the south of European Russia [13].

#### Record in European Russia

A single female specimen of *Medythia nigrobilineata* was collected by sweep netting on wasteland with grasses on May 19, 2016, at Imereti Resort, Adler District, Sochi (43°25′ N, 39°59′ E) [13].

#### Biology

Ogloblin [44] and Koyama [29, 43, 45] describe the biology of *M*. *nigrobilineata* in its native range. *Medythia nigrobilineata* develops only on soybeans. Adults feed on leaves and often damage immature pods. Moreover, beetles can feed on leaves of rice and sugarcane. Adults hibernate among fallen leaves and in the soil. In spring, they begin to feed on soybean seedlings and damage leaves. Females lay eggs in the soil. Larvae feed on root nodules and pupate in the soil.

#### Vector of dispersal

The specimen was collected 4 km from the international airport at Sochi. We suspect that *M*. *nigrobilineata* was unintentionally introduced from Asia through this airport.

#### Invasion status

Although we found only one specimen, it likely represents a population (at least temporal). First, the likelihood of collecting an individual in nature from a current introduction rather than from a breeding population is vanishingly small. Second, the establishment of *M*. *nigrobilineata* in Krasnodar Krai is possible, because soybean is widely cultivated in this region [46]. Special surveys of soybean plantations are necessary to reveal whether the pest is established.

#### Economic impact

Adults and larvae of *M*. *nigrobilineata* are serious pests of soybeans in China, Japan and the Russian Far East [29, 44, 47, 48]. Thus, the establishment of this species in the Krasnodar Region poses a serious threat to soybean production.

### 2013 –*Chrysolina americana* (Linnaeus, 1758) (Chrysomelinae)

#### Native range

**Fig 5:**
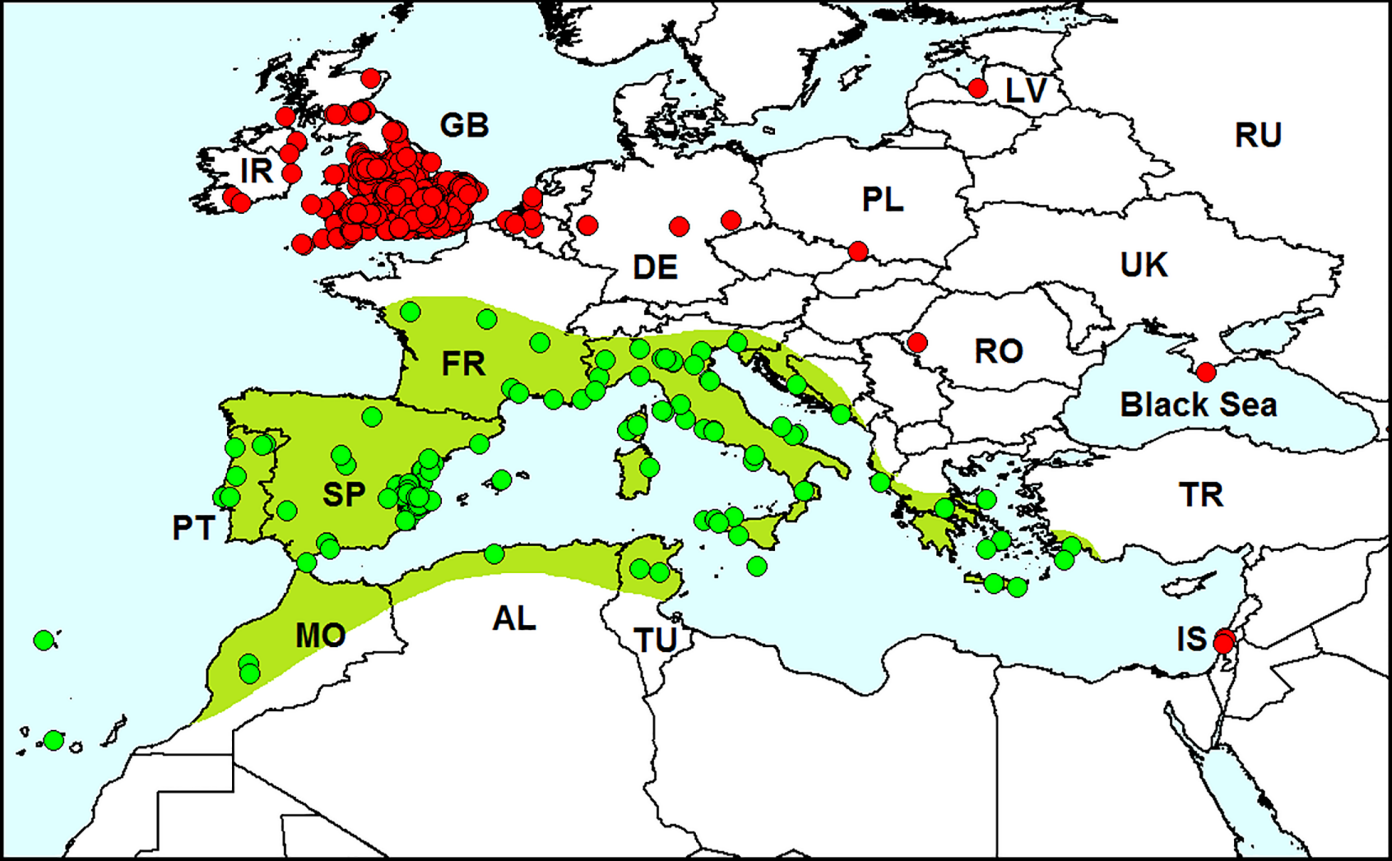
Distribution of *Chrysolina americana*. *Red dots*–localities in which *Ch*. *americana* was recorded outside the native range; *green dots* on the *green background–*localities in the native range. Database on localities of *Chrysolina americana* is provided in S1 Appendix.

Rosemary beetle is native to Mediterranean countries: Albania, Croatia, France, Greece, Italy, Malta, Portugal, Slovenia, Spain, Serbia, Macedonia, Algeria, Morocco, Tunisia and Turkey (Fig 5) [6, 19]. In particular, *Ch*. *americana* occurs on islands: Mallorca, Corsica, Sardinia, Cyclades, Crete, Madeira, north Aegean Islands and Malta [49].

#### Invasion history

Before the 1990s, specimens of *Ch*. *americana* were collected several times outside the native range: in 1936 and 1938 in Belgium [50]; before 1950 in Romania, Austria and Germany (examined specimens from MTD); and in 1963 in the United Kingdom [51]. However, these findings most likely reflected only temporary populations. When *Ch*. *americana* was again recorded from the United Kingdom in the 1990s, MacLeod [52] supposed that it would not establish because of the cold climate. However, notably, *Ch*. *americana* established and began to spread quickly and is now common throughout the United Kingdom and Ireland [50]. Introduction of the beetle was also reported in several other European countries and Israel (Table 1).

**Table 1:** Records of *Chrysolina americana* outside the native range.

Invaded regions	Years of records	Sources of information
United Kingdom	1963, 1994–2017	[50–52]
Poland	<2011	[53]
Latvia	1996	[54]
Israel	2014, 2015, 2016	[55]
Belgium	1936, 1938, 2016, 2017	[6, 50]
Netherlands	1995, 2005, 2017	[6, 50, 56]
Germany	<1950, 2008, 2017	Examined specimens from MTD and [49, 50]
Switzerland	<2017	[49]
Ireland	2012, 2013, 2015	[50]
Romania	<1950	Examined specimens from MTD and [57]
Crimea	2013	Examined specimens from NO

At present, the species has spread and is established throughout the Netherlands; however, not on native plants. *Chrysolina americana* thrives in gardens on rosemary and lavender and on cultivated *Salvia* but has not been recorded on native *Salvi*a species in the wild [56]. Records of *Ch*. *americana* from Poland, Romania, Germany and Switzerland are supposedly questionable because of the Mediterranean origin of the species [49, 53, 57]. We suppose that these records indicated cases of introduction of the species outside the native range. It is difficult to determine which records indicate establishment of species and which represent translocations or temporal populations.

#### Record in Crimea

*Chrysolina americana* has not been recorded from Russia or the Ukraine until now. Here, we present the first record of *Ch*. *americana* on the Crimean Peninsula: 13 specimens of this species were collected in Crimea in 2013 (Yalta, Bakhchisaray highway, sanatorium "Uzbekistan," 225 m a.s.l., on *Rosmarinus officinalis*, 01.06.2013, leg. N.V. Okhrimenko).

#### Biology

Adults and larvae feed on leaves of plants of the family Lamiaceae: *Rosmarinus officinalis*, *Lavandula spp*., *Salvia spp*., *Thymus spp*., *Perovskia atriplicifolia* and others [55].

#### Vector of dispersal

Because *Rosmarinus* and *Lavandula* are popular garden plants throughout Europe, *Ch*. *americana* has been translocated outside the native range along with its host plants [6]. Opinions about natural dispersal abilities of *Ch*. *americana* are contradicting. MacLeod [52] states that *Ch*. *americana* is flightless and therefore is restricted in its dispersal abilities. However, Beenen and Roques [6] indicate that this species has good flight capacities and disperses naturally by flight. Webster et al. [58] suppose that expansion of the rosemary beetle to the United Kingdom has been expedited by climate change.

#### Invasion status

Whether *Ch*. *americana* is established in Crimea or only a temporal population was recorded is not clear. Further observations are necessary to answer this question, because the example of the population in the United Kingdom shows that this species is able to establish quickly and become a serious invasive pest.

#### Economic impact

Rosemary beetle is a garden pest and damages the foliage and flowers of various aromatic plants including lavender, rosemary and sage [55]. These plants are widely cultivated in Crimea. Thus, if *Ch*. *americana* becomes abundant, the beetle can cause negative economic consequences in the region.

### 2011 –*Epitrix hirtipennis* (Melsheimer, 1847) (Alticinae)

#### Native range

**Fig 6:**
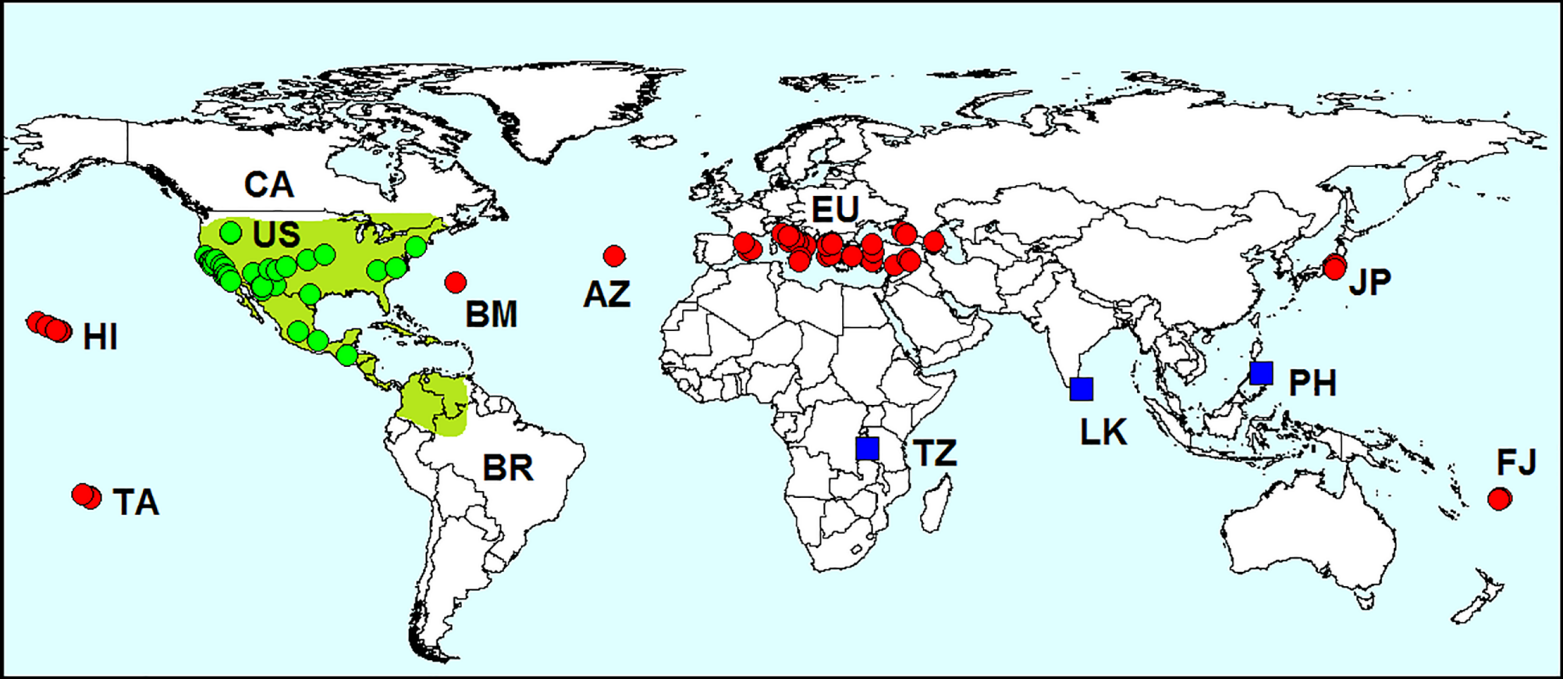
Geographical distribution of *Epitrix hirtipennis*. *Red dots*–localities outside the native range; *green dots* on the *green background*–localities in the native range; *blue squares*–questionable records. AZ–Azores, BM–Bermuda, BR–Brazil, CA–Canada, EU–Europe, HI–Hawaii, FJ–Fiji, JP–Japan, LK–Sri Lanka, PH–Philippines, TA–Tahiti, TZ–Tanzania, US–United States. Sources of information about records in the native range were examined specimens from VNIIKR, ZIN and the literature [61–64]. Sources of information about records outside the native range are indicated in Table 2. Database on localities of *Epitrix hirtipennis* is provided in S1 Appendix.

Tobacco flea beetle is native to the south of North America, north of South America and the Caribbean Islands (Fig 6) [59, 60].

**Table 2:** Records of *Epitrix hirtipennis* outside the native range.

Invaded regions	Years of records	Sources of information
Hawaii	1892, 1893, 1927, 1942, 1960, 1961, 1962, 1967, ~1979, ~2002	[64–68]
Bermuda	<1924, 1969	[69, 70]
Tahiti	1973, 1974, 1977, 1978	[67]
Fiji	1978	[64, 67, 71]
Azores	1984	[72]
Mainland Italy	1983, 1990	[73–75]
Greece	1988, 2004	[76–77]
Turkey	1993, 2010, 2013, 2015, 2016	[78–82]
Macedonia	1996, 2011, 2013	[83–85]
Albania	<1997	[85]
Balearic Islands	1998, 2016	[27]
Bulgaria	<2000	[86, 87]
Sicilia	2000, 2010	[88]
Syria	2002	[89]
Japan	2011, 2016	[90, 91]
Russia	2011, 2013, 2016	[11, 92] and original data
Georgia	2014	[93]
Croatia	<2015	[94]
Mainland Spain	2015	[39]
Philippines (questionable record)	<1987	[75, 95]
Sri Lanka (questionable record)	<1923	[61, 75]
Tanzania (questionable record)	2013	[96]

#### Invasion history

**Fig 7:**
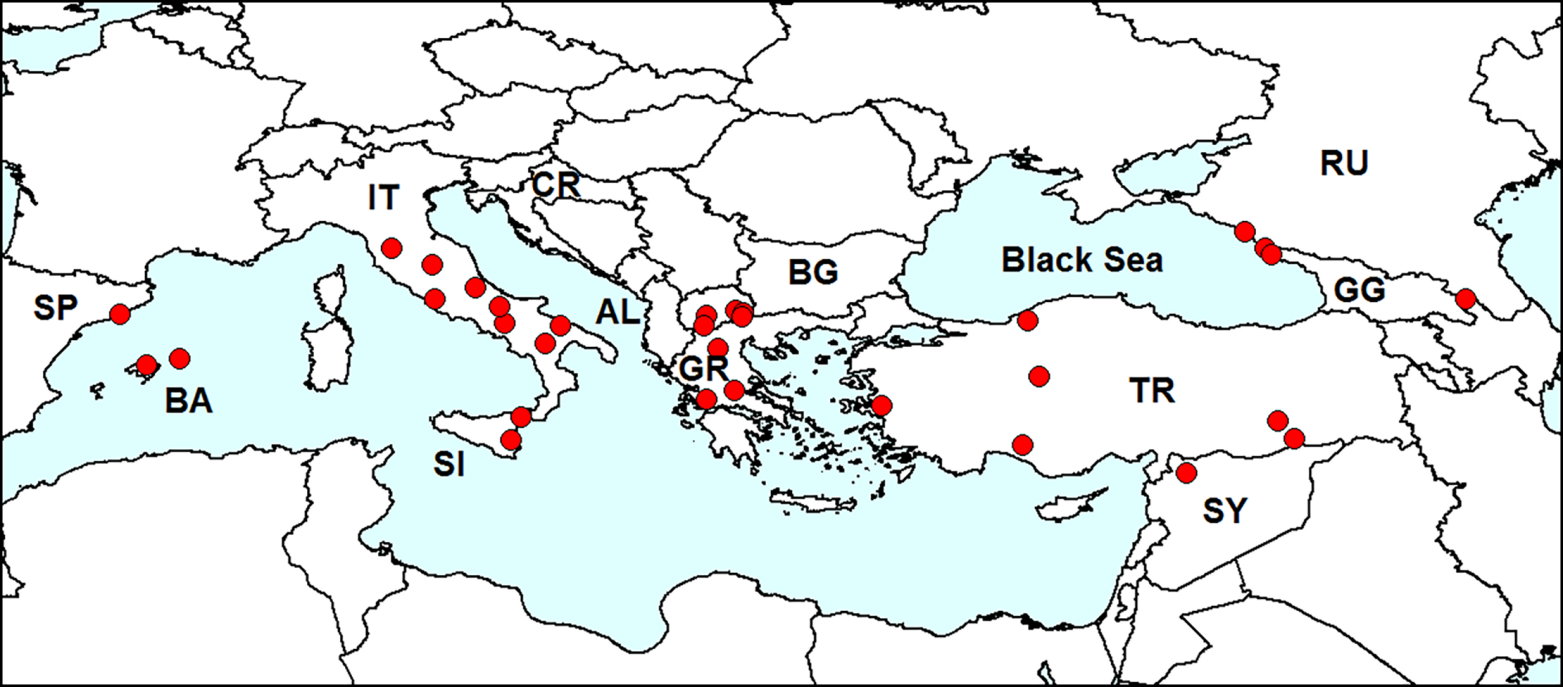
Distribution of *Epitrix hirtipennis* in Europe. *Red dots*–localities in Europe. IT–Italy (first record in 1983), GR–Greece (1988), TR–Turkey (1993), AL–Albania (<1997), BA–Balearic Islands (1998), SY–Syria (2002), RU–Russia (2013), GG–Georgia (2014), CR–Croatia (<2015), SP–mainland Spain (2015). Sources of information are indicated in Table 2. Database on localities of *Epitrix hirtipennis* is provided in S1 Appendix.

*Epitrix hirtipennis* began to spread outside the native range at the end of the 19th century (Figs 6 and 7; Table 2). First, the beetle was introduced to islands in Atlantic and Pacific oceans: Hawaii, Bermuda, Tahiti, Fiji and Azores, and has become common there.

In 1983, this species was found in Europe for the first time in northern Italy [62] and was the first alien flea beetle introduced to Europe. The beetle then spread to south and central Italy, Greece, Turkey, Spain, Macedonia, Bulgaria, Syria, European Russia and Georgia. In 2011, *E*. *hirtipennis* was found to be common on Honshu (Japan) [90]. In some reviews, records from Sri Lanka [61], the Philippines [95, 75] and Tanzania [96] are mentioned; however, these records are doubtful, because no references to the source of information are given.

#### Records in European Russia

In 2011, *E*. *hirtipennis* was first recorded in Russia, namely, on a tobacco plantation in Krasnodar City (south in European Russia) [92]. In 2013, 2016 and 2018, we found *E*. *hirtipennis* on the Black Sea coast, namely, in Tuapse and Sochi (Khosta District and Adler District, Imereti Resort) [11]. We collected five specimens in these localities in ruderal vegetation (see S1 Appendix).

#### Biology

Adults feed on leaves of plants of the family Solanaceae. Larvae develop on roots. Bieńkowski and Orlova-Bienkowskaja [97] review the host plants of *E*. *hirtipennis* in different regions. In Italy, *E*. *hirtipennis* has been observed to shift onto native Solanaceae [5].

#### Vector of dispersal

Since *E*. *hirtipennis* was the first alien flea beetle introduced to Europe, its record on the continent puzzled experts in Chrysomelidae. *Epitrix hirtipennis* was assumed to have arrived in Europe as aerial plankton with easterly trade winds blowing from the New World to Europe [98]. However, we believe that the most likely vector of dispersal is an unintentional introduction of larvae in soil with imported planting material.

#### Invasion status

*Epitrix hirtipennis* is established in the region. Localities of the records in Europe and Asia indicate that the species is dispersing eastward.

#### Economic impact

This species is known primarily as a pest of tobacco but can also feed on eggplant, potato, tomato and many other solanaceous plants [99].

#### Remark

The representatives of the genus *Epitrix* are particularly prone to invasions. Five species are established outside their native ranges in other continents and on islands. One additional representative of the genus, *Epitrix setosella* (Fairmaire, 1888), was reportedly introduced outside the native range, namely, from east Asia to Georgia [93]. However, our examination of the specimens identified as "*E*. *setosella"* from the collection of G.O. Japoshvili showed that the identification was incorrect and that those specimens belonged to *E*. *pubescens* (Koch, 1803).

### 2011 –*Diabrotica virgifera* LeConte, 1868 (Galerucinae)

#### Native range

The western corn rootworm *Diabrotica virgifera* originates from the New World. The initial range is Mexico or Central America [100]. Now, the range in the Americas includes Canada, Costa Rica, Guatemala, Mexico, Nicaragua and the USA [4].

#### Invasion history

**Fig 8:**
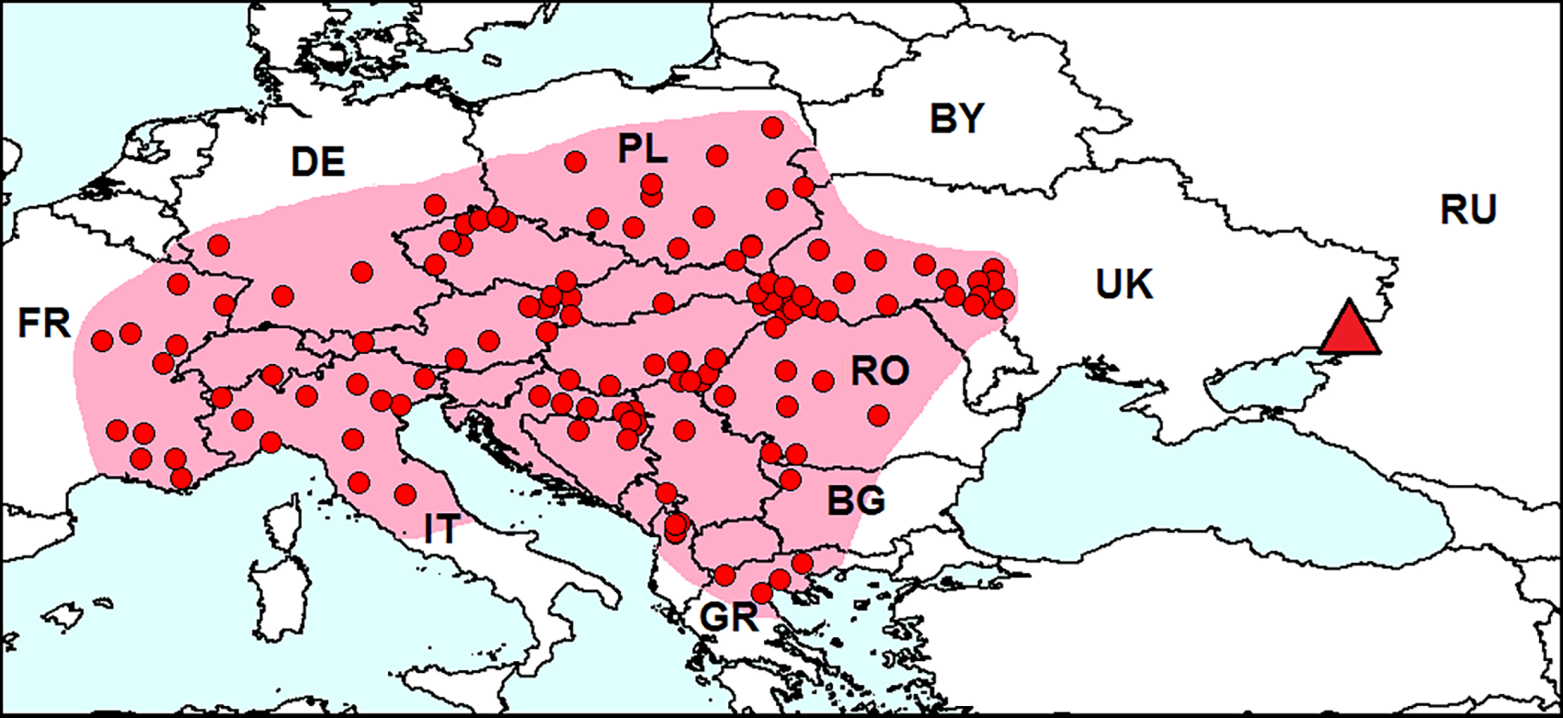
Distribution of *Diabrotica virgifera* in Europe. *Red dots* on the *pink background*–localities of species in the secondary range. *Red triangle*–locality of the interception of one specimen in Russia in 2011. BG–Bulgaria, BY–Belarus, DE–Germany, FR–France, PL–Poland, RO–Romania, RU–Russia, UK–Ukraine. Database on localities of *Diabrotica virgifera* is provided in S1 Appendix.

Man has greatly increased the range of this species in the Americas by the cultivation of corn [100]. In Europe, *D*. *virgifera* was first observed near the Belgrade airport, Serbia, in 1992 [101]. After several introduction events to different regions of Europe [102], the species has become widespread. Now, the beetle is recorded in at least 22 European countries (Fig 8) [4].

#### Record in European Russia

In 2011, *D*. *virgifera* was captured with a pheromone trap at the border of Russia in the Rostov Region near the international highway [10].

#### Biology

Adults of *Diabrotica virgifera* feed on leaves, silks, pollen, and young kernels of corn, and larvae develop on roots [103].

#### Vector of dispersal

*Diabrotica virgifera* has been translocated from North America to Europe several times in aircraft laden with goods and materials [102]. The beetles fly well, and therefore, they spread in Europe both by hitchhiking and naturally [104].

#### Invasion status

*Diabrotica virgifera* has apparently not established in Russia yet, because it was intercepted only once in 2011.

#### Economic impact

*Diabrotica virgifera* is a major pest of cultivated corn. Feeding on the root system, larvae cause most of the damage. The species is included on the A2 List of quarantine pests of EPPO [4].

### 1984 –*Phyllotreta reitteri* Heikertinger, 1911 (Alticinae)

#### Native range

**Fig 9:**
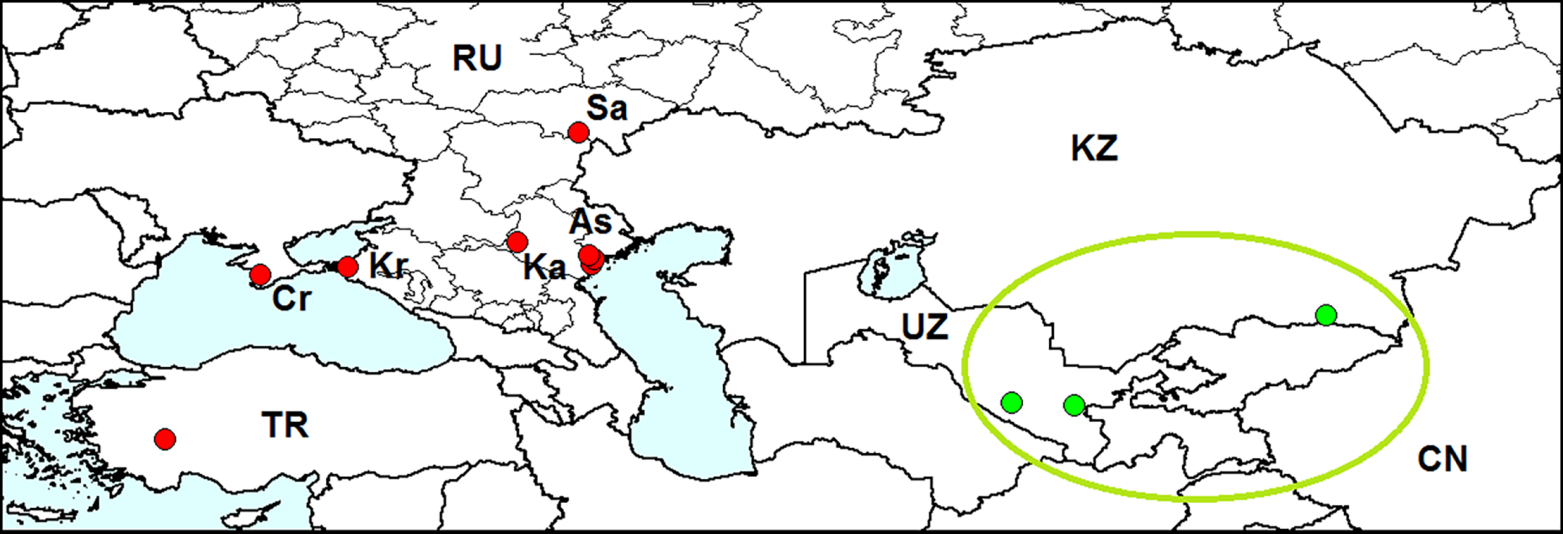
Distribution of *Phyllotreta reitteri*. *Green dots*–localities in the native range; *red dots*–localities outside the native range (records after 1984). CN–China, KZ–Kazakhstan, RU–Russia, TR–Turkey, UZ–Uzbekistan, As–Astrakhan Region, Cr–Crimea, Kr–Krasnodar Territory, Ka–Kalmykia, Sa–Saratov Region. Sources of information are indicated in Table 3.

We believe that the native range of *Ph*. *reitteri* is in central Asia (Fig 9), because the primary host plant *Lepidium latifolium* originates from that region [105]. Before the 1980s, *Ph*. *reitteri* was recorded in Kazakhstan and Uzbekistan only [106–108]. Most likely the recent record from west China [109] also belongs to the native range.

#### Invasion history

In 1984, *Ph*. *reitteri* was first found outside the native range, namely, in Crimea [110]. Then, in 1986–2012, the beetle was found in four regions in the south of European Russia (Table 3) and in 2002 in Turkey [111].

**Table 3:** Records of *Phyllotreta reitteri* outside the native range.

Invaded regions	Years of records	Sources of information
Crimea: Simferopol	1984	[110] and examined specimens from SM
Kalmykia: Elista, Lagan and Dzhalykovo	1986, 2011, 2012	Our collection and examined specimens from GK
Astrakhan Region: Liman	2010	Our collection
Saratov Region: D'yakovka	2004	Examined specimens from AU
Krasnodar Territory: Temryuk and Golubitskaya	2007, 2008, 2010	Examined specimens from ZIN (collected by B.A. Korotyaev and A.G. Moseyko)
Turkey: Denizli	2002	[111]

#### Distribution in European Russia

*Phyllotreta reitteri* occurs in Kalmykia, Saratov Region, Astrakhan Region and Krasnodar Territory (see Table 3).

#### Biology

Experiments conducted in cages and in the field show that the primary host plant of *Phyllotreta reitteri* is the perennial pepperweed *Lepidium latifolium* [112]. Adults feed on leaves, and larvae mine petioles and shoots. This plant of Asian origin has been cultivated as a spice and vegetable since the twelfth century [113]. Now, pepperweed is widespread in Asia and Europe, has been recorded on all continents and has become an invasive weed in North America [112]. In European Russia, *Ph*. *reitteri* occurs in moist habitats, such as on the banks of rivers and ponds and in irrigated parks.

#### Vector of dispersal

Unknown.

#### Invasion status

We believe that *Ph*. *reitteri* is alien in European Russia. The species meets at least four criteria of alien beetle species [97] as follow:

1. Detection of an established population of the species, which was not recorded earlier. *Phyllotreta reitteri* appeared outside the historical range (central Asia) in the 1980s.
2. Disjunction of the range, which cannot be explained by disjunction of suitable landscapes or host plant ranges. The range of *Ph*. *reitteri* consists of two parts: one is in central Asia, and the second is in the south of European Russia, the Crimean Peninsula and Turkey. The distance between these two parts is more than 1500 km.
3. Expansion of a part of the range isolated from the main part. Records in Turkey and Astrakhan and Saratov regions in the 2000s most likely indicate the expansion of the range.
4. Feeding on an alien host plant. *Lepidium latifolium* originates from central Asia [105].

It is unlikely that the species occurred in the region but remained unnoticed for a long time, because this beetle is large for the genus *Phyllotreta* and has characteristic bright coloration. No collected specimens of *Phyllotreta reitteri* from the south of European Russia were found in the rich collection of the Zoological Institute of the Russian Academy of Sciences, although the collection contains thousands of specimens of other *Phyllotreta* species collected in this region at the end of the 19th and the first half of the 20th century.

#### Economic impact

*Phyllotreta reitteri* is regarded as a potential biological control agent of perennial pepperweed [105].

### 1982 –*Zygogramma suturalis* (Fabricius, 1775) (Chrysomelinae)

#### Native range

Ragweed leaf beetle is native to the USA and the south of Canada [59].

#### Invasion history

**Fig 10:**
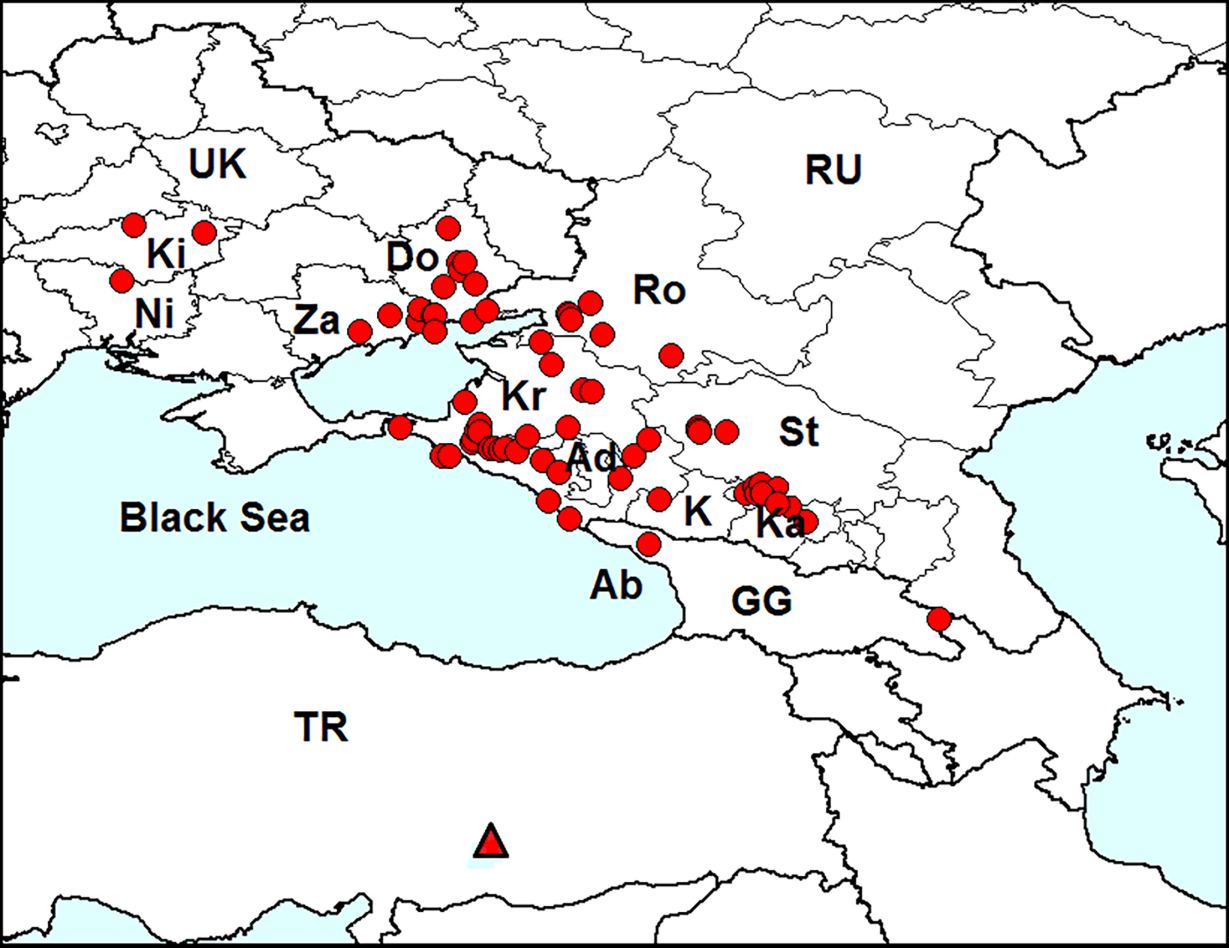
Distribution of *Zygogramma suturalis* in Europe. *Red dots*–localities of species. GG–Georgia, RU–Russia, TR–Turkey, UK–Ukraine, Ab–Abkhazia, Ad–Adygea, Do–Donetsk Region, K–Karachay-Cherkessia, Ka–Kabardino-Balkaria, Ki–Kirovograd Region, Kr–Krasnodar Territory, Ni–Nikolaev Region, Ro–Rostov Region, St–Stavropol Territory, Za–Zaporizhje Region. Database on localities of *Zygogramma suturalis* is provided in S1 Appendix.

*Zygogramma suturalis* (Fig 10) was introduced to the USSR from Canada and the USA for control of one of the most noxious invasive weeds *Ambrosia artemisiifolia* [114]. The beetles were released in 16 provinces of the USSR: in European Russia, the Ukraine, Georgia, Kazakhstan and the Far East. The most intensive work was performed in the south of European Russia, primarily in the Rostov Region and in Stavropol and Krasnodar territories [114]. The first release (1500 specimens) was performed in the vicinity of Stavropol in 1978. In 1981–1983, *Z*. *suturalis* became abundant and began to spread quickly. Now, the beetle is relatively widespread in the south of European Russia and occurs also in the southeast of the Ukraine and in Georgia [115]. In 1985, one specimen was found in Turkey, but the species did not establish, with no other further records [116, 117].

*Zygogramma suturalis* when introduced to the south of European Russia showed rapid evolutionary changes in flight capacity (development of flight ability and morphological changes) within only five generations [118]. The morphological changes were so significant that the new subspecies *Zygogramma suturalis volatus* Kovalev, 2002 was described [118].

In 1982–1985, beetles from Stavropol Territory were released in Primorje (Far East). In the 1990s, the population of *Z*. *suturalis* in the Far East had supposedly disappeared. However, in 2010, this species was re-discovered in the region, although it was not abundant [119]. *Zygogramma suturalis* was also released in Croatia and Australia but failed to establish, whereas releases of *Z*. *suturalis* in China in 1985 resulted in establishment in some locations [109]. Record of *Z*. *suturalis* from Kazakhstan [109] most likely refers to the releases rather than to established populations. There are no current records of *Z*. *suturalis* from this country [120].

#### Distribution in European Russia

Now, *Z*. *suturalis* occurs in five provinces in the south of European Russia: Stavropol Territory, Krasnodar Territory, Adygea, Rostov Region, Karachay-Cherkessia [121, 122] and Kabardino-Balkaria (see S1 Appendix).

#### Biology

Larvae and adults feed on leaves, shoots and inflorescences of *Ambrosia artemisiifolia* [8]. The species is very abundant in some localities, occurs in river valleys, on the fringes of forests, and in saline areas [123].

#### Vector of dispersal

The beetle was intentionally introduced for biological control of *Ambrosia artemisiifolia*.

#### Invasion status

*Zygogramma suturalis* is established in European Russia.

#### Economic impact

Soon after the introduction of *Z*. *suturalis* to the Caucasus, the density of some populations was as high as 100 million specimens per km^2^. *Zygogramma suturalis* completely destroyed *Ambrosia* in some locations. However, 10 years after the introduction, surveys revealed that although *Z*. *suturalis* remained abundant in some places, the beetle did not significantly affect the general density of the host plant. The “plant-phytophagous” system reached an equilibrium [122, 124]. The same situation is observed in the Far East [119].

### 1958 –*Leptinotarsa decemlineata* (Say, 1824) (Chrysomelinae)

#### Native range

The Colorado potato beetle originates from the central highlands of Mexico [125].

#### Invasion history

**Fig 11:**
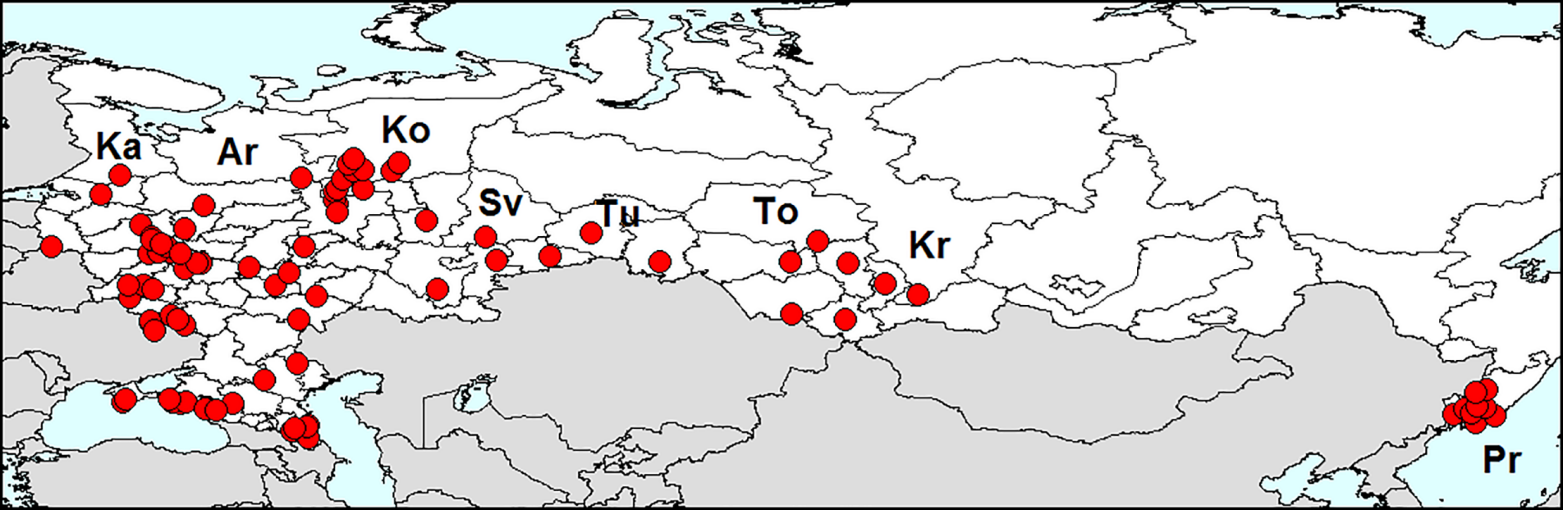
Distribution of *Leptinotarsa decemlineata* in Russia. Ar–Arkhangelsk Region, Ka–Karelia, Ko–Republic of Komi, Kr–Krasnoyarsk Territory, Pr–Primorsky Territory, To–Tomsk Region, Tu–Tumen Region. Database on localities of *Leptinotarsa decemlineata* is provided in S1 Appendix.

From the beginning of the 19th to the beginning of the 20th century, *L*. *decemlineata* colonized all of North America [7, 125]. In 1922, *L*. *decemlineata* was found established in Bordeaux (France) and began to spread in Europe. Now, it is distributed almost throughout Europe and northern Asia [4, 9, 19, 125]. Presently, the Colorado potato beetle damages potato crops all over Europe, Asia Minor, Iran, central Asia, and western China [125]. The general distribution of *Leptinotarsa decemlineata* is well known [4]; thus, we present the map of distribution of this species in Russia only (Fig 11).

#### Expansion to Russia

In 1958, expansion of the pest range reached the western border of the USSR [9]. Now, the Colorado potato beetle is common all over European Russia, even in the north: in the Leningrad Region [9] and the Republic of Komi [126]. The range has expanded to most parts of Siberia. The northern boundary of the area passes through Karelia, Arkhangelsk Region, Republic of Komi, Tumen Region, Tomsk Region and Krasnoyarsk Territory. Since 2000, an isolated part of the range is also in the Far East in Primorsky Territory [9].

#### Biology

In European Russia, *L*. *decemlineata* feeds on *Solanum tuberosum*, *S*. *lycopersicum*, *S*. *melongena*, *S*. *laciniatum*, *S*. *dulcamara*, *Hyoscyamus niger* and *Atropa belladonna* [9, 28, 127]. The beetle occurs not only in agricultural landscapes but also in undisturbed natural communities, in particular, on riverbanks [3].

#### Vector of dispersal

This pest enters new territories primarily because of unintentional transportation with potato and self-dispersal of beetles sometimes aided with the winds [7].

#### Economic impact

*Leptinotarsa decemlineata* is the most devastating pest of potato and other cultivated plants of the family Solanaceae [125].

## Discussion

Analysis of invasions of leaf beetles to European Russia revealed some general tendencies. First, recent translocations and establishment of Chrysomelidae species outside their native ranges are obviously much more common than previously. Only three alien leaf beetle species were recorded in European Russia in the 20th century: *Leptinotarsa decemlineata*, *Phyllotreta reitteri* and intentionally introduced *Zygogramma suturalis*. However, in 2000–2017, as many as six alien species were found in the region. This situation corresponds to the general tendency of exponential increase in the rate of invasions of leaf beetles to Europe [6] and the increase in invasions of beetles feeding on living plants [128]. The current situation reflects the increase in international trade, particularly the trade of living plants and the increase in air transport. In most cases, leaf beetles are translocated to remote regions with imported plants or (as in the case of *Diabrotica virgifera*) by hitchhiking in airplanes [102, 104]. Most of the alien leaf beetles are associated with agricultural plants, but some species (for example, *Phyllotreta reitteri*) develop on weeds.

After establishment outside their native ranges, leaf beetles can quickly spread by unintentional introduction by man and natural dispersal. For example, *Diabrotica virgifera* was first recorded in Europe in 1992 but has now spread to at least 21 countries becoming a major pest of corn in some regions [4]. *Luperomorpha xanthodera* has occupied all of Europe from Spain to Russia in only 15 years. Therefore, the records of new alien pest leaf beetles are very important and should be published quickly.

In some cases, invasions of leaf beetles are unpredictable. For example, establishment of *Leptomona erythrocephala* native to Spain in the Caucasus is rather surprising. However, in other cases, emergence of alien species is easy to predict. For example, it was obvious that *Epitrix hirtipennis* could appear in the south of European Russia, because the range in Europe was expanding to the east. Similarly, in the early 20th century, it was obvious that *Leptinotarsa decemlineata* would appear in Europe because of its outbreak and quick range expansion in North America. In general, when the range of some leaf beetle species is quickly expanding, or when the species has been recorded established somewhere outside the native range, this species should be regarded as a potential invader worldwide. For example, global invasion of *Epitrix hirtipennis* began from its establishment in Hawaii at the end of the 19th century [65].

Leaf beetles can be potentially translocated from any part of the world. Four species alien to European Russia originate from North America, two from the Mediterranean region and three from Asia. Recently, the invasions of beetles of east Asia are becoming more important [128]. Establishment and quick spread of *Luperomorpha xanthodera* in Europe reflects this current tendency.

The Black Sea region is more prone to invasions of leaf beetles than other regions of European Russia. The many records of alien species in this region could not be explained by more intensive survey, because we have the database of localities of leaf beetles collected in all regions of European Russia (approximately 30 000 localities) [129]. The many invasions in the Black Sea region correspond to the general tendency of many invasions of leaf beetles in territories with a warm and wet climate. For example, Italy occupies first place in Europe for the number of alien leaf beetles [6].

However, predicting whether an introduced species can become established based on a simple comparison of climate in native and invaded ranges is difficult (or even impossible), because the establishment of a species depends on a complex of interacting abiotic, biotic and anthropogenic factors. For example, when *Ch*. *americana* was recorded for the second time in the United Kingdom in the 1990s, MacLeod [52] supposed that the beetle would not establish because of the cold climate; however, *Ch*. *americana* has established and become a common pest.

Alien leaf beetles can spread to native communities and become naturalized. For example, *Zygogramma suturalis* also occurs in native, undisturbed communities of nature reserves [130, 131]. Usually, alien leaf beetles remain strictly related to their original, alien plants [5, 6]. This relation is also true for the Chrysomelidae of European Russia. However, some species can also feed also on native plants or cultivated plants from other regions. In particular, *Leptinotarsa decemlineata* feeds not only on cultivated plants but also on *S*. *dulcamara* and *Hyoscyamus niger*. When a species is established in native communities and feeds on native plants, it is fully naturalized, i.e., ecologically undistinguishable from native species. Because of full naturalization, the distinction between native species and those established before the 20th century is difficult.

Because the invasions of leaf beetles can cause tremendous economic consequences, and because of the exponential increase in the rate of invasions recently observed, special attention should be paid to the study of these invasions. Monitoring is necessary to reveal the cases of emergence of species outside their native ranges. General trends of invasions of leaf beetles should be examined and analyzed carefully.

## Supporting information

S1 Appendix Localities of species mapped in the article. (XLSX)

S2 Appendix Adult of *Leptomona erythrocephala* collected in Russia. (JPG)
