# Supplementary figures and images for: Alien leaf beetles (Coleoptera, Chrysomelidae) of European Russia and some general tendencies of leaf beetle invasions
Source: PLoS One. 2018 Sep 7;13(9):e0203561. doi: 10.1371/journal.pone.0203561 (PMC6128575; doi:10.1371/journal.pone.0203561)

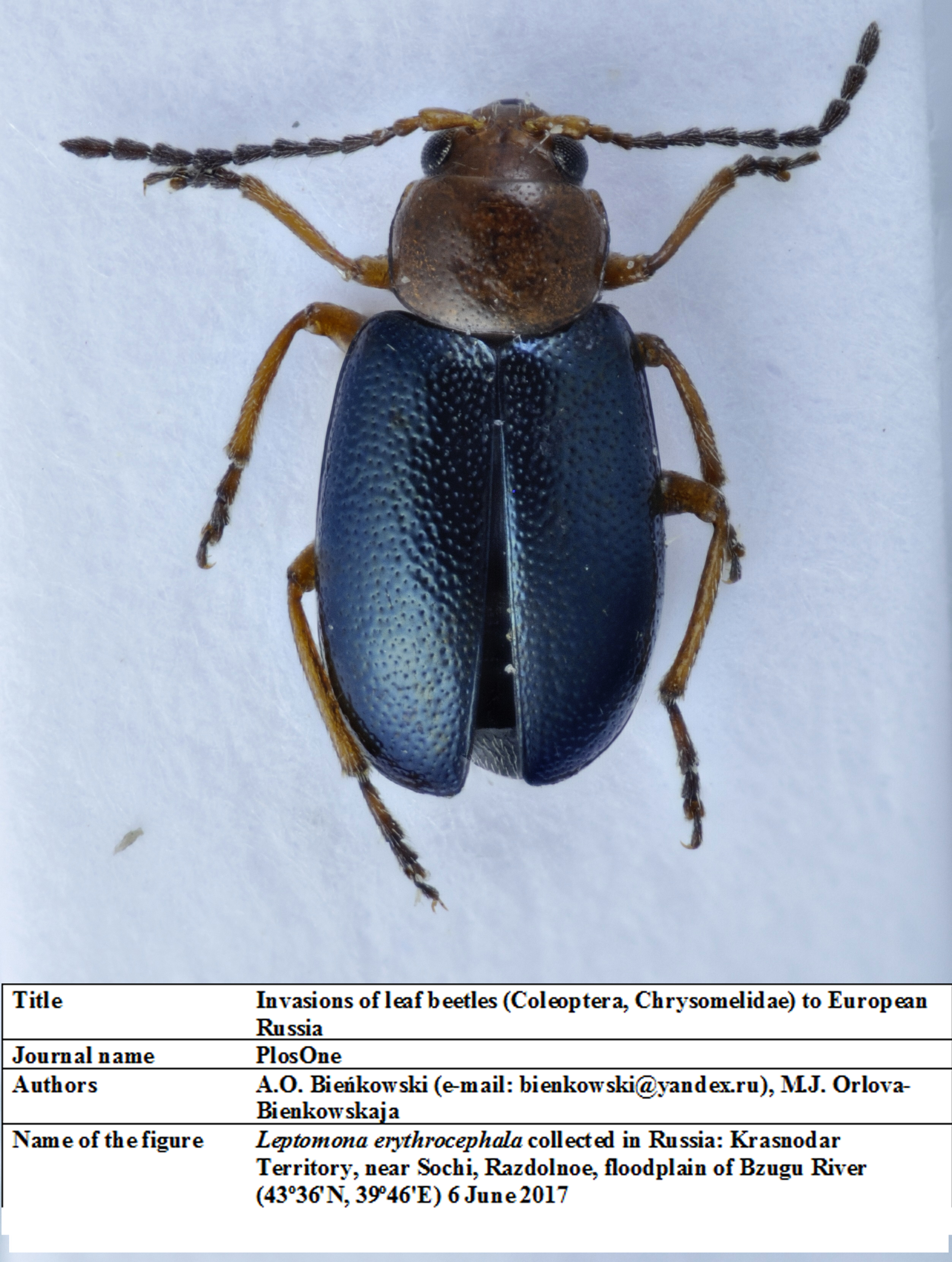

Supplement: S2 Appendix — (JPG) [file pone.0203561.s002.jpg]
